# Betulin Acid Ester Derivatives Inhibit Cancer Cell Growth by Inducing Apoptosis through Caspase Cascade Activation: A Comprehensive In Vitro and In Silico Study

**DOI:** 10.3390/ijms24010196

**Published:** 2022-12-22

**Authors:** Paweł Pęcak, Marta Świtalska, Elwira Chrobak, Grzegorz Boryczka, Ewa Bębenek

**Affiliations:** 1 Department of Organic Chemistry, Faculty of Pharmaceutical Sciences in Sosnowiec, Medical University of Silesia in Katowice, 4 Jagiellońska Str., 41-200 Sosnowiec, Poland; 2 Institute of Immunology and Experimental Therapy, Polish Academy of Sciences, 12 Rudolfa Weigla Str., 53-114 Wrocław, Poland; 3 Department of Gastroenterology and Hepatology, Faculty od Medical Sciences, Medical University of Silesia, 14 Medyków Str., 40-752 Katowice, Poland

**Keywords:** betulin, anticancer activity, apoptosis, caspase 3/7, lipophilicity, ADME

## Abstract

Betulin, or naturally occurring triterpene, possesses promising antiproliferative activity. To further explore this potential, thirty-eight betulin acid ester derivatives modified at the C-28 position were tested for antitumor activities. Four human cancer cell lines, MV4-11 (leukemia), A549 (lung), PC-3 (prostate), MCF-7 (breast) as well as the normal BALB/3T3 (mouse fibroblasts) cell line were examined using MTT and SRB assays. A few derivatives exhibited strong antiproliferative activity with IC_50_ values between 2 and 5 µM. Subsequent mechanistic studies revealed that some derivatives induced apoptosis by inducing caspase-3/7 activity. A strong structure–activity correlation of tested compounds has been proposed along with experimental and in silico pharmacokinetic properties.

## 1. Introduction

Cancer is one of the most serious threats to public health worldwide. After cardiovascular diseases, it is second-leading cause of mortality [1]. Chemotherapy agents still remain the key method to combat cancer, but developing resistance to currently used anticancer drugs forces the search for new agents with diverse mechanisms of action [2].

Chemical compounds isolated from plants are one of the richest sources of new drug candidates and are widely used in many different areas of pharmacotherapy [3,4]. Abundant in many plants, triterpene, also called betulin **1** is an example of such a compound. It possesses anticancer, antiviral, antibacterial and anti-inflammatory properties [5,6,7,8,9]. High natural availability and ease of obtaining as well as broad biological activity makes it a good candidate for further research as a potential drug.

It has been shown that betulin **1** selectively affects various cancer cells and is a low-toxic compound towards normal cells. It inhibits proliferation of more than 20 different cancer cell lines. Li et al. showed that betulin **1** induces apoptosis in human cancer cells such as HeLa (cervix carcinoma), HepG2 (hepatoma), A549 (lung adenocarcinoma), MCF-7 (breast cancer), SK-HEP-1 (hepatoma), PC-3 (prostate carcinoma), NCI-H460 (lung carcinoma) and K562 (erythroleukemia) [10]. These activities against a broad spectrum of different cell lines undergo many different mechanisms of action, which include apoptosis induction, inhibition of cell viability and metastasis, interference with angiogenesis and differentiation, change in signal transductions or DNA synthesis [11]. In addition, it was observed that depolarization of the mitochondrial membrane potential and mitochondrial release of cytochrome c followed the betulin **1** treatment. Moreover, betulin-induced apoptosis was mediated by sequential activation of caspase-9 and caspase-3/-7 and cleavage of poly(ADP-ribose) polymerase (PARP) [10].

**Figure 1:**
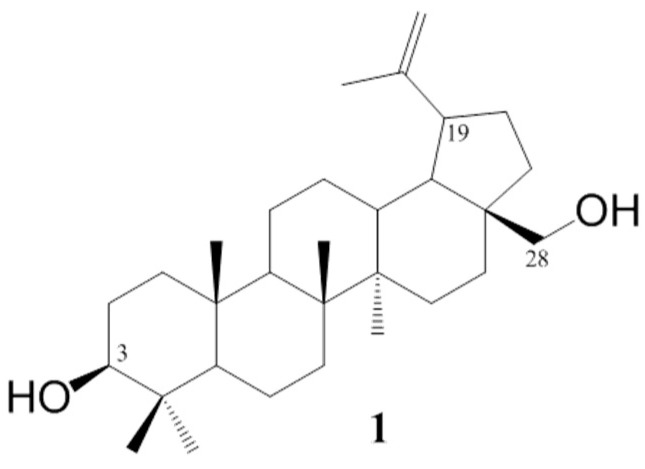
Chemical structure of betulin.

The betulin **1** chemical structure contains two hydroxyl groups at carbons C–3 and C–28 and an ispropenyl moiety attached to carbon C–19 (Figure 1). These moieties allow for chemically modifying the betulin **1** structure to enhance its biological activity. The use of standard methods of chemical synthesis and biocatalytic processes allowed for obtaining a number of active pentacyclic triterpene derivatives [12,13].

A broad spectrum of derivatives such as esters [14], amides [15] and conjugates [16] exhibited a very good 1–5 µM range of anticancer activity potential against most clinically important cancers. To better understand their antitumor activity, a number of the molecular mechanisms of action studies have been conducted. A vast majority of them have shown that the induction of apoptotic cell death is an essential mechanism of anticancer agents’ activity [8,15,17].

Yang et al. obtained a group of six betulin acid ester derivatives and demonstrated their high antiproliferative activity with an apoptosis-induction mechanism [15]. In continuation of an ongoing program aiming at developing novel anticancer drugs, we decided to explore this small, but promising group of betulin **1** derivatives. Considering already described, potent pharmacophore groups, we have significantly widened the panel of derivatives by introducing new moieties [6]. Simultaneously with already published antiviral studies [6], and previously untested in the context of cytotoxic activity, compounds were tested for their antiproliferative activity against four cancer lines and one normal cell line. Considering the high cell line and mechanism specificity of active betulin derivatives and to verify if a previously described mechanism was retained in new compounds, we performed an induction of apoptosis analysis. Additionally, to widen and further explore the possible mechanistic pathway of tested derivatives, a caspase-3/7 and cell cycle arrest assays were conducted. Furthermore, to assess the potential of active compounds as potential drugs, an experimental and in silico analyses of lipophilicity and pharmacokinetic parameters were conducted. Finally, to facilitate the further search for new derivatives from the group of derivatives, the activity–structure relationship was proposed.

## 2. Results and Discussion

### 2.1. Preparation and Structure of Betulin Derivatives

**Scheme 1:**
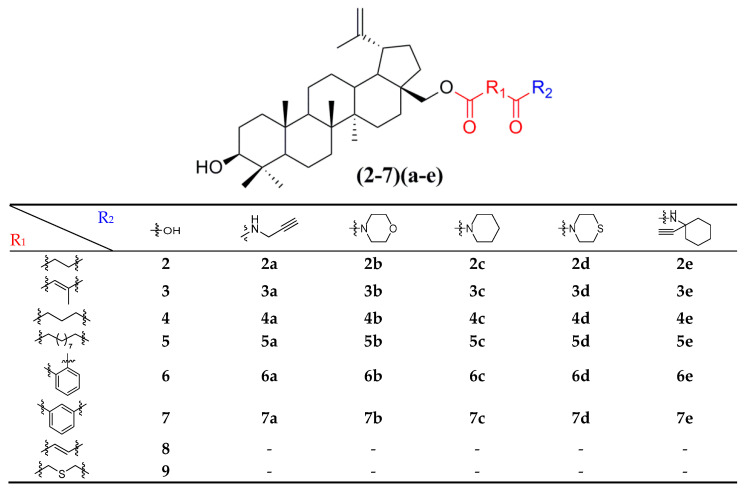
Structures of betulin derivatives used.

Thirty-eight betulin acid ester derivatives were synthetized, as published previously [6]. Briefly, in the first step, the 28-hydroxyl group of betulin was esterified with various dicarboxylic acids (**R_1_**) to produce esters **2**–**9**. Next, derivatives **2**–**7** were acylated using five different amines (**R_2_**) to produce amides (**2**–**7**)(**a**–**e**). The wide variety of substituents, including those containing heteroatoms, aliphatic, aromatic, linear and cyclic systems, as well as peer-to-peer synthesis, allows for the precise correlation of biological activity with the diverse structures of derivatives. The structures of tested compounds have been presented in Scheme 1.

### 2.2. Antiproliferative Activity

#### 2.2.1. Overview of Cell Lines and Compounds Tested

The in vitro antiproliferative activity tests have been conducted against four human cancer cell lines: MV4-11 biphenotypic B myelomonocytic leukemia cell line, A549 lung carcinoma cell line, MCF-7 breast carcinoma cell line, PC-3 prostate carcinoma cell line and one normal BALB/3T3 mouse fibroblast cell line. To preliminarily determine the active compounds, all derivatives were tested against the MV4-11 cell line. Based on these results, ten of the most active amide derivatives (half minimum inhibitory concentration, IC_50_ < 10 µM; compounds: **2(a**–**d)**, **3b**, **3d**, **4a**, **4b**, **4d**, **5b**), four dicarboxylic acid precursors (**2**, **3**, **4**, **5**) as well as doxorubicin and betulin **1** as positive controls were chosen for further studies against remaining cell lines. All tested compounds were dissolved in DMSO and diluted with a culture medium to a desired concentration. Four piperidine derivatives (**3c**, **4c**, **5c**, **7c**) were not soluble in DMSO; ergo, studies were not conducted on them. Cell viability, after 72 h incubation of the cells with tested compounds, was determined using tetrazolinum dye MTT (for MV4-11 cells) or sulforhodamine B (SRB test, for MCF-7, A549, PC-3, BALB/3T3 cells) colorimetric assays.

#### 2.2.2. Initial Screening and Activity against MV4-11 and BALB/3T3 Cell Lines

Thirty-five compounds were tested in vitro against the MV4-11 leukemia cell line and compared with doxorubicin, cisplatin and betulin **1** as positive controls. Betulin’s half minimum inhibitory concentration (IC_50_ = 18.16 µM) was preconceived as a cutoff value for active compounds. The results, presented in the form of a heatmap, are shown in Table 1. The color scheme visualizes active compounds in green and inactive ones in red. Color saturation corresponds to their degree of activity as indicated on the scale.

The results show several strong structure–activity relationships. Briefly, almost all succinic (**2 a**–**d**), citraconic (**3 a,b,d**), glutaric (**4 a,b,d**) as well as two azelaic (**5 a**–**b**) acid amides show three to eleven-fold better activity than their parent ester compounds. In the case of succinic acid analogs, the **2a** and **2d** IC_50_ values were comparable to cisplatin (2.03, 3.16 and 1.83 µM, respectively). These findings correlate well with previous studies covering 28 substituted betulin derivatives containing a terminal triple bond as well as morpholine moieties [15,18]. Several other amides (**2b**, **2c**, **3d**, **5b**, **4b**, **4a**, **4d**) and azelaic acid ester **5** show IC_50_ between 3.94 and 7.04 µM. The presence of a terminal carboxylic group seems to be necessary for ester activity, as single carboxylic acid analogs of **5** do not exhibit activity at all [19].

A small, aliphatic linker such as succinic and glutaric tends to provide better activity with short, acyclic amines such as propargylamine. On the other hand, bigger or cyclic linkers such as phthalic, citraconic or azelaic tend to favor cyclic amines such as piperidine, morpholine or thiomorpholine. Interestingly, all m-phthalic and 1-ethynylcyclohexylamine derivatives were shown to be inactive compared to their precursors and reference compounds. In all cases, the activity drops dramatically from two to more than fifty-fold. These observations are consistent with previous studies [15]. The introduction of rigid aromatic or aliphatic rings with no heteroatoms may lead to reduced water solubility as well as cell membrane penetration, and thus degradation of activity [14,20], although, is it unclear why m-phthalic derivatives are less active than phthalic ones. Moreover, the activity of amides decreases with the elongation of a dicarboxylic acid linker and in the presence of aromatic rings in the following order: succinic > glutaric > citraconic > azelaic > phthalic > m-phthalic.

To acquire more in-depth insight into structure–activity relationships, it is crucial to compare these results with activity against normal, non-cancer cell lines such as BALB/3T3 mouse fibroblasts. The cytotoxicity data has been presented as a heatmap analogous to the MV4-11 results (Table 2). As expected, the antiproliferative activity of the tested compounds against normal cell lines was lower than that of the tumor one. The IC_50_ of positive controls betulin and doxorubicin was approximately 2 and 18 times lower than in the MV4-11 cell line, respectively. Preliminary analysis of the obtained data shows that structure–activity relationships are more dependent on the amines used rather than dicarboxylic linkers. The morpholine derivatives **2b**, **3b** and **5b** with IC_50_ 25.04, 24.69 and 20.36 µM were 3 to 6 times less active compared to MV4-11. Interestingly, propargylamine derivatives **2a** and **4a** almost retained their MV4-11 activity and were much less susceptible to cytotoxic agents than the BALB/3T3 cell line. Contrariwise, the thiomorpholine derivatives **2d**, **3d** and **4d** lost their activity completely, beyond the maximum tested concentration used (50 µM).

#### 2.2.3. Activity against A549, MCF-7 and PC-3 Cell Lines

After preliminary MV4-11 cell line antiproliferative screening, selected compounds (**2**, **2**(**a**–**d**), **3**, **3b**, **3d**, **4**, **4a**, **4b**, **4d**, **5**, **5b**) were tested on A549, MCF-7 and PC-3 cell lines to evaluate their influence (after 72 h of incubation) on cancer cells with diverse etiology (Table 3, Table 4 and Table 5). By comparing doxorubicin results, the susceptibility of cell lines increases in the following order PC-3 (IC_50_: 0.43 µM) < BALB/3T3 (IC_50_: 0.18 µM) < MCF-7 (IC_50_: 0.08 µM) < A549 (IC_50_: 0.04 µM) < MV4-11 (IC_50_: 0.01 µM). In the case of betulin **1**, the sequence is: MCF-7 (IC_50_: 38.82 µM) < PC3 (IC_50_: 32.46 µM)~BALB/3T3 (IC_50_: 31.79 µM) < MV4-11 (IC_50_: 18.16 µM) < A549 (IC_50_: 15.51 µM). Betulin **1** shows a relatively small two-fold range from minimal to maximal IC_50_ values, whereas for doxorubicin, this parameter equals 43. Briefly, the A549 cell line shows similarly high susceptibility as leukemia MV4-11; on the other hand, MCF-7 and PC-3 cell lines are less leverageable and similar to the BALB/3T3 cell line. The structure–activity relationships of MCF-7 and PC-3 lines tend to promote morpholine moiety (**b**) with all tested dicarboxylic acid linkers (IC_50_ for MCF-7 12.27–21.61 µM and 17.86–24.84 µM for PC-3) over similar thiomorpholine ones (**d**, IC_50_ > 46.11 in all cases). This tendency also occurs in the case of the A549 cell line, but with lower IC_50_s (8.03–12.58 µM for morpholine derivatives (**b**) and 6.78–32.01 µM for thiomorpholine derivatives (**d**)).

#### 2.2.4. Cross Cell Lines Summary of Antiproliferative Activity

The large variety of tested compounds and cell lines allows for a broad look at antiproliferative activity. The five cell lines studied can be divided into two groups because of their susceptibility to the tested compounds and structure–activity relationships. The first group contains A549 and MV4-11 cell lines and presented high susceptibility to the applied compounds. The second group, containing remaining lines MCF-7, PC-3 and BALB/3T3, exhibited very similar IC_50_ and structure–activity relationships.

Within all cancer cell lines, morpholine amides presented consistently high activity. Unfortunately, high activity against normal Balb/3T3 cell line results in a relatively low selectivity index (Table 6). Similarly, succinic-propargylamine derivate **2a** has been found to be most active against MV4-11, PC-3 and BALB/3T3 cell lines. On the other hand, thiomorpholine amides of succinic (**2d**) and citraconic (**3d**) acid showed SI values of 15.8 and 11.8, respectively; therefore, in combination with one of the highest IC_50_ values (3.16 and 4.23 µM, respectively), this constitutes a solid basis that thiomorpholine derivatives are a promising group of anti-leukemia compounds and should be subject to further studies. Other derivatives exhibit selectivity indexes generally around or above 1, with an exception across all cell lines to propargylamine **2a** and **4a** compounds (ranges from 0.1 to 0.7). In the case of A549, all active derivatives (**2b**, **2c**, **3b**, **3d**, **4b**, **5**) exhibited SI in a range from 3.1 to 7.4. For MCF-7 and PC-3 lines, SI fluctuates around 1.

In all cases, dicarboxylic esters were less active than betulin **1**, except azelaic ester, which was more potent from 1.35 to 3.62-fold. Interestingly, the betulin **1** to **5** IC_50_ ratio was almost similar in the case of A549, MCF-7 and MV4-11 (3.16, 3.16 and 3.62, respectively) and PC-3 and BALB/3T3 (1.64 and 1.35, respectively). Furthermore, almost in all cell lines, azelaic ester **5** transformed into morpholine amide **5b,** resulting in a decrease of activity, which is the opposite of previous morpholine-derivatives correlations.

### 2.3. Mechanistic Studies

#### 2.3.1. Induction of Apoptosis

**Figure 2:**
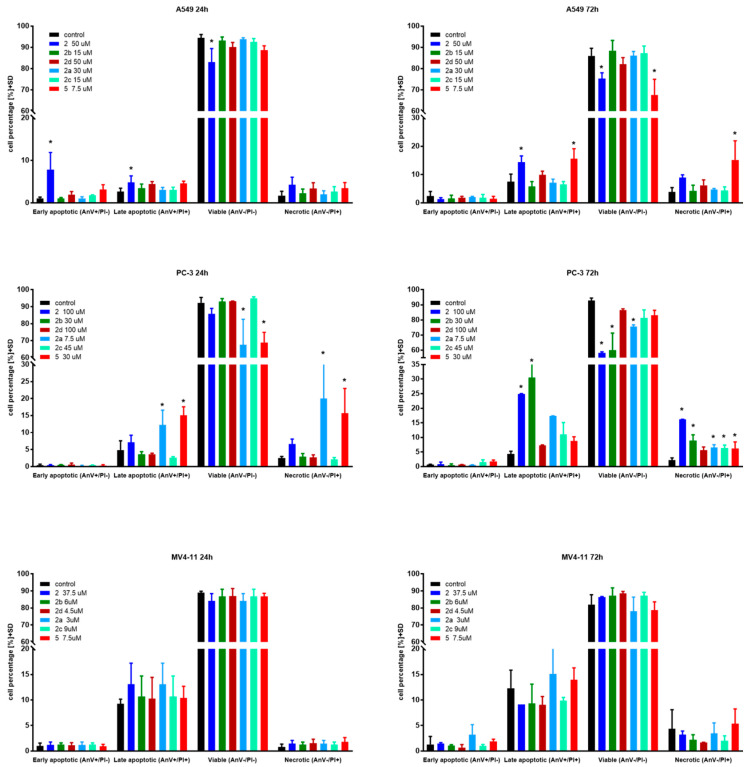
Induction of apoptosis by tested compounds after 24 and 72 h. * statistically significant versus control cells, *p* < 0.05. Compounds were used in concentrations of about 1.5 × IC_50_.

Apoptosis is one of a cell’s natural mechanisms for programmed cell death. It plays a crucial role in homeostasis by eliminating the unwanted or unnecessary cells. One of the most important functions of apoptosis is prevention of cancer. However, cancer cells often lose the ability to enter the path of apoptosis, which allows them to survive longer and grow in an uncontrolled manner. Regardless of that, apoptosis still remains the most important molecular mechanism to combat cancer cells; therefore, research is conducted to obtain agents with the ability to selectively induce apoptosis in cancer cell lines [21]. Previous research on betulin **1** and its derivatives revealed that apoptosis is a key mechanism of antitumor action of this group of compounds [10,15,22]. Based on that, to further evaluate the mechanism of action of obtained derivatives, we conducted apoptosis induction tests using the Annexin V (AnV)/Propidium iodide (PI) staining method after 24 and 72 h of incubation with tested compounds in about 1.5 × IC_50_ concentration. Results are presented in Figure 2.

In the case of the A549 lung carcinoma cell line after 24 h incubation time, one compound **2** showed significantly increased levels of early-apoptotic and apoptotic cells at the expense of the living cell population. As expected, longer treatments shifted the ratio towards apoptotic and necrotic cells. Additionally, after 72 h incubation time, compound **5** exhibited significantly upraised levels of apoptotic and necrotic cells compared to the control. A similar effect was observed on the PC-3 cell line after 24 h incubation with derivatives **2**, **2a** and **5**. After 72 h incubation time, almost all compounds caused a significant uprising in the number of necrotic cells. Compounds **2** and **2b** at PC-3 cells exhibited a similar profile as **5** at A549 after 72 h incubation time by shifting the ratio of the living cell population towards apoptotic and necrotic ones. The tested compounds did not influence apoptosis on the MV4-11 leukemia cell line, which may suggest a different mechanism of action.

Overall, apoptosis induction activity was exposed by succinic acid ester **2**, its propargylamine **2a** and morpholine **2b** amides as well as azelaic acid ester **5**.

#### 2.3.2. Cell Cycle Arrest

**Figure 3:**
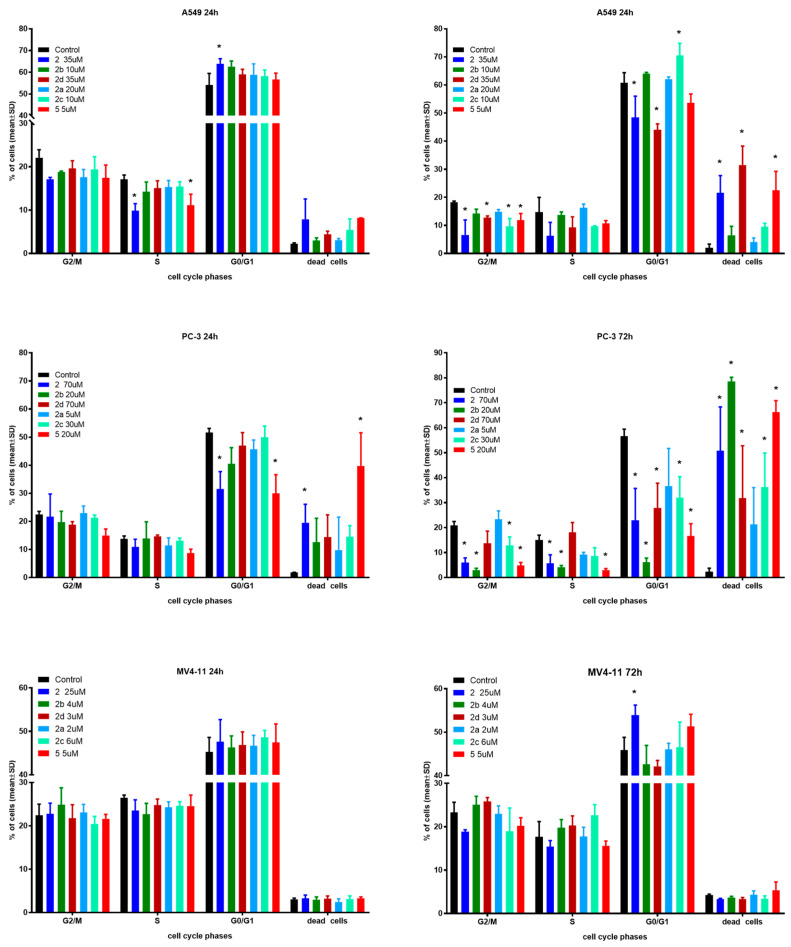
Influence of tested compounds on cell cycle after 24 and 72 h incubation. * statistically significant versus control cells, *p <* 0.05. Compounds were tested in concentrations of about 1 × IC_50_.

In the next step of mechanistic evaluation, the cell cycles of MV4-11, PC-3 and A549 cells were analyzed after 24 and 72 h of exposure to tested compounds in their IC_50_ concentrations (Figure 3).

The effects of the selected derivatives on the cell cycle were analyzed by flow cytometry using PI staining.

In the case of A549 cells after 24 h incubation time, only derivative **2** showed significantly induced cell cycle arrest in the G0/G1 phase compared to the control. Compounds **2** and **5** reduced the number of cells in the S phase with an increased number of dead cells. After 72 h incubation time, compounds **2**, **2d** and **5** caused a greater percentage of dead cells with a decrease in G2/M and G0/G1 phases. Furthermore, compound **2c** induced cycle arrest in the G0/G1 phase with a decrease in the G2/M phase.

The PC-3 cell line showed a similar tendency to A549 cells. A longer incubation time results in a higher percentage of dead cells, but this effect is much stronger compared to A549 cells. Compound **5** resulted in 80% dead cells versus 15% after the same 24 h incubation time used in the studies.

The MV4-11 cell cycle was generally not affected by the tested compounds in both incubation times. Only compound **2** exhibited a statistically significant increase in the number of cells in the G0/G1 phase compared to the reference, which resulted in a lower number of cells in the G2/M and S phases. There were no differences between the two incubation times used in the studies.

Regardless of the type of cell line, the obtained results do not indicate any influence on nucleic acid synthesis (S) and the G2/mitosis steps of cell cycle. As expected, longer incubation with more cytotoxic compounds resulted in a higher percentage of dead cells, which suggests time-dependent antiproliferative activity. When compared to control cells, only PC-3 cells after 72 h incubation time exhibited a high shift towards dead cells. The cell cycle arrest in the G0/G1 phase for some compounds mentioned earlier is in line with previous reports on the effect of betulin derivatives on tumor cell cycles [11,17,22].

#### 2.3.3. Caspase-3/7 Activity

**Figure 4:**
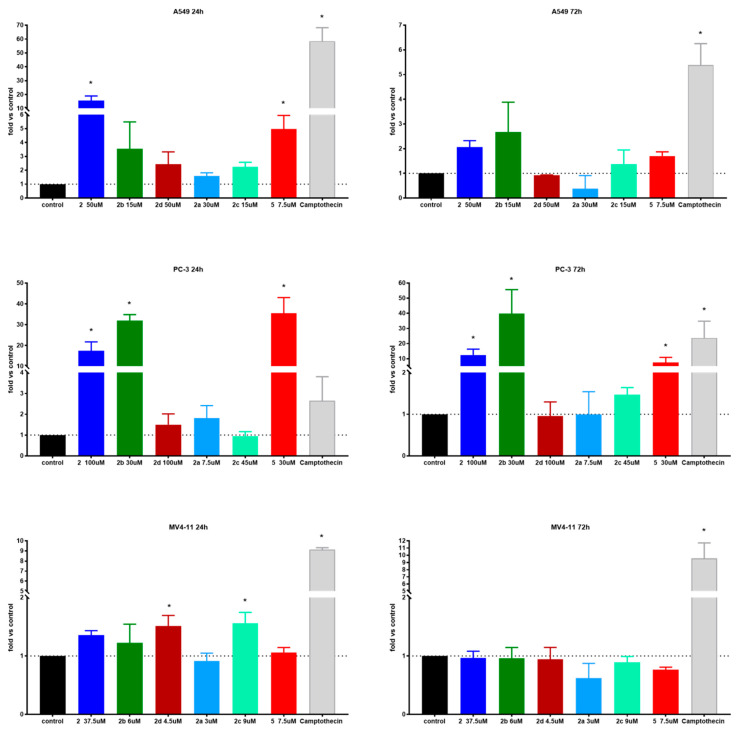
Caspase-3/7 activity of tested compounds after 24 and 72 h. * statistically significant versus control, *p <* 0.05. Compounds were tested in concentrations of about 1.5 × IC_50_.

Caspase-3 and caspase-7 are cysteine-aspartic acid proteases that can directly execute apoptosis followed by sequential activation of caspase-8 or caspase-9. Activation of these enzymes leads to apoptotic cell death; thus, measuring their activity is a good method to determine the pro-apoptotic potential of tested compounds. One of the mechanisms of betulin’s antiproliferative activity is activation of these enzymes; therefore, to further examine the mechanism of action of tested derivatives, we decided to determine their levels [10]. In the current study, the selected derivatives have been tested after 24 and 72 h of incubation time with 1 × IC_50_ (data not showed) and 1.5 × IC_50_ concentrations. Results are presented in Figure 4.

The A549 lung carcinoma cell line presented hugely elevated caspase 3/7 activity after treatment with succinic acid ester **2** and its two amides **2c**, **2b** and azelaic ester **5** as well. As expected, extending of the incubation time from 24 to 72 h led to a several-fold decrease in activity, but was still statistically up-leveled. Caspases-3/7 are executioner enzymes, so as the percentage of dead cells increases, their activity also declines. In almost all cases, the 1.5 × IC_50_ compound concentration was needed to induce statistically important level increases. Interestingly, in the case of ester **5** and amide **2b** and **2c** derivatives, the caspase-3/7 elevated activity correlates well with a high degree of antiproliferative activity. On the other hand, succinic acid ester **2** shows highest caspase 3/7 activity and one of the lowest antiproliferative activities (Table 7). This might suggest a different mechanism of action for these derivatives.

The succinic acid derivatives had a similar influence on prostate carcinoma cell line PC-3 as of A549. After 24 h, the incubation time levels of caspase-3/7 were 2 to 35 times higher than the control. Contrary to MV4-11 cell line results, caspase levels were 3 to 15 times higher than positive control camptothecin. Activity was retained at a longer incubation time, but compared to camptothecin, it dropped below 1 one. The morpholine ester of **2**–**2b** exhibited a very high level of caspase-3/7 after 24 and 72 h time at 1.5 × IC_50_ concentration. The activity of **5** was very high at shorter incubation times and dropped significantly later. The concentration-dependent increase of caspase-3/7 activity in the PC-3 cell line as well as the key role of these enzymes in apoptosis has been described before [23,24].

In the case of the MV4-11 leukemia cell line, only compounds **2c** and **2d** after 24 h exhibited increased levels of caspase-3/7. After 72 h, no effect on caspases was observed at any concentration used. The MFC-7 breast carcinoma cells do not express caspase-3/7 and, therefore, studies were not conducted on them [25].

### 2.4. Lipophilicity and ADME Properties

Lipophilicity is an important physicochemical parameter that determines the possibility of transporting substances through biological membranes. This descriptor is related to processes such as absorption and distribution, as well as the excretion of xenobiotics in the body. Lipophilicity also affects the ability of a compound to bind to plasma proteins and form a compound–receptor complex. The pharmacodynamic and pharmacokinetic properties of medicinal substances often correlate with their lipophilicity [26].

Among the different methods used to determine the lipophilicity parameter, in this work, we used the experimental reverse phase thin layer chromatography (RP-TLC) method as well as in silico calculation methods. The retention mechanism in the RP-TLC method is based on the partition of substances between two phases. Silica gel (RP-18 F_254S_ plates) was used as the hydrophobic stationary phase. In contrast, the hydrophilic mobile phase was a binary mixture of acetone and an aqueous solution of TRIS buffer. The chromatographic analysis by RP-TLC is presented in the Supplementary Materials. The chromatographic parameters (R_M0_, log P_TLC_) determined for the carboxyacyl **2**–**9** and amide (**2**–**7**)(**a**–**e**) betulin derivatives are presented in Table 8. The literature lipophilicity (logP_lit_) and experimental (R_M0_) values of reference compounds are presented in Table S1.

The values of the log P_TLC_ of the compounds studied were in the range of 6.796–8.861. The propargylamine-containing derivative **2a** was characterized by the lowest value of experimental lipophilicity (log P_TLC_ = 6.796) and the highest activity against MV4-11, Balb/3T3 and PC-3 cells (IC_50_: 2.03, 3.10 and 4.47 µM, respectively) among all tested derivatives. The results indicated that introducing morpholinyl moiety derivatives (**2**–**5**)**b** led to the reduction of lipophilicity. The morpholine-derivatives **6b** and **7b** showed higher values of log P_TLC_ than compounds (**2**–**5**)**b,** which is due to the presence of an aromatic ring in their chemical structure. The amides containing phthalic and isophthalic moieties **7**(**a**, **c**–**e**) were inactive against cancer cell lines tested and had high experimental lipophilicity values (log P_TLC_ = 7.972–8.284). It was also shown that compounds (**2**–**6**)**e** containing the cyclic 1-ethynylcyclohexylamine substituent had high values of the experimental lipophilicity parameter ranging from 8.205–8.861. This group also includes compound **6e** with the highest log P_TLC_ value among all of the studied betulin dicarboxylic acid ester derivatives.

**Figure 5:**
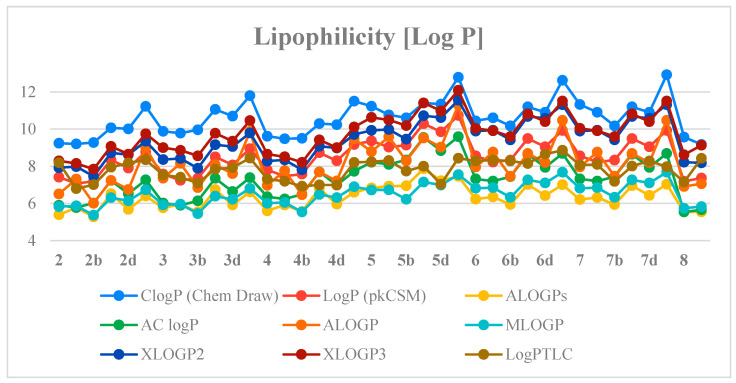
The profile of change for calculated and experimental lipophilicity of the studied compounds.

Next, the previously determined experimental lipophilicity of the tested derivatives was compared with the theoretical values. The theoretical values of the partition coefficients (log P) were derived from eight calculation programs (C log P (Chem Draw Ultra 14.0, http://www.perkinelmer.co.uk/category/chemdraw), Log P (pkCSM), ALOGPs, AC log P, ALOGP, MLOGP, XLOGP2 and XLOGP3) [27,28]. The obtained results (Table S2, Supplementary Materials) show that compound **2a** has the lowest value of the experimental and theoretical lipophilicity parameters. Determined theoretical values of log P for the tested compounds are in the range of 5.28–12.93. Analysis of the data obtained shows that the highest log P values are from C log P (Chem Draw) and the lowest from ALOGPs. Table S3 (Supplementary Materials) presents the correlation matrix for the theoretical parameters of lipophilicity of the compounds. Obtained correlation coefficients are in the range of 0.791–0.981. The highest correlation value was recorded for Log P (pkCSM) and AC log P (0.981). Figure 5 shows a comparison of the experimental lipophilicity values (log P_TLC_) for all derivatives with the corresponding values obtained from theoretical calculations.

The study of biological activity of the synthesized compounds is complemented by evaluation of pharmacokinetic parameters such as drug similarity, ADME profile (Absorption, Distribution, Metabolism, Excretion) and toxicity. To assess the potential of the chemical compound to be active via oral administration, the criteria formulated by Lipinski and referred to as the Rule of Five are most often taken into account. Lipinski’s rule is based on four physicochemical parameters such as molecular weight (MW ≤ 500), lipophilicity (LogP ≤ 5), the number of hydrogen bond donors (nHBD ≤ 5) and acceptors (nHBA ≤ 10) [29]. These parameters are related to intestinal permeability and water solubility [30]. The calculated pharmacokinetic properties of the tested compounds are shown in Table 9.

The molecular weights of the tested compounds are in the range of 540.79–718.12 and are higher than the value adopted by Lipinski (MW ≤ 500), determining a chemical compound with good oral bioavailability. Furthermore, for the most active compound **2a**, both the molecular weight (MW = 579.87) and the experimentally determined value of the lipophilicity parameter (Table 8, logP_TLC_ = 6.796) violate this rule. The high molecular weight and higher lipophilicity of the compound result in poor water solubility and, therefore, low oral bioavailability. Oral drug delivery is the most common mode of administration and thus approximately 60% of the small-molecule drugs known on the market are used. Nevertheless, there is an increasing number of potential synthetic drug candidates that exhibit poor water solubility [31,32]. Various methods of orally formulating drugs with poor water solubility have been developed to overcome this limitation. These include crystal engineering methods (formation of metastable polymorphs or co-crystals), chemical modifications (conversion into prodrugs or salts), particle size reduction (micronization and nanoparticles) or amorphization (formation of solid dispersions). Another way is to use drug carrier systems such as micelles, nanoparticles, cyclodextrins and lipid-based preparations (e.g., liposomes) [31].

Taking into account another criterion for assessing drug-likeness, known as the Weber rule, a molecule of an orally administered drug should contain less than 10 rotating bonds (nROT ≤ 10) and its polar topological surface should be less than 140 Å^2^ (TPSA ≤ 140 Å^2^) [33]. Only **5–5e** series compounds have a higher number of rotational bonds, and all other compounds satisfy this rule (Table 9).

A method for predicting intestinal permeability involving both passive and active absorption mechanisms of a new potential drug candidate is to use the Caco-2 model [34]. For the tested compounds, the Caco-2 perm values calculated by the pkCSM server are in the range of 0.461–0.736, which indicates their poor intestinal absorption [35]. The correlation between the molecular descriptors, anticancer activity and the R_M0_ values for betulin derivatives are presented in the Tables S4 and S5.

In general, the results suggest a strong inversely proportional correlation between compound lipophilicity and anticancer activity. The rule of five turned out to be unrelated to the activity of the tested compounds, as all of them do not satisfy the criteria, but still exhibit activity.

## 3. Conclusions

The search for new anticancer drugs is currently one of the main objectives of medicinal chemistry. After that, understanding of the mechanism of action of potential anti-cancer drugs is one of the most important steps in the advancement of chemotherapy of these diseases. In this paper, we have significantly widened and explored a new panel of betulin acid ester derivatives against existing and previously undescribed human cancer and normal cell lines. Furthermore, we have validated previous findings relating to their proapoptotic mechanisms of action. Considering the vast panel of possible mechanisms of actions of betulin derivatives, additional cell cycle arrest and caspase-3/7 activity assessments have been done.

Overall, derivatives with succinic acid linker **2**(**a**–**d**) as well as morpholine amine (**2**–**4**)**d** exhibited very good cytotoxic activity. Thiomorpholine derivatives (**2**–**4**)**d** are good candidates for further studies, as they showed very good selectivity indexes and level of activity as well. On the other hand, all m-phthalic and 1-ethynylcyclohexyl derivatives were shown to be inactive. Subsequently, apoptosis detection and cell cycle analysis indicated induction of apoptosis as the main mechanisms of action of active compounds. With the exception of the leukemia MV4-11 cell line, proapoptotic activity correlates well with an increased level of apoptosis executioner enzymes–caspases-3/7. To complete the characterization of compounds, lipophilicity analysis linked logP_TLC_ values with degree of antiproliferative activity.

The obtained results confirm the dependence of the activity and mechanisms of action of particular betulin derivatives on the cell line used, and at the same time, the need to study them for individual compounds. Additionally, the study presented strong findings that were consistent with the literature and the structure–activity relationships, which provide a solid basis for further studies on betulin derivatives as potent anticancer agents.

## 4. Materials and Methods

### 4.1. Compounds

Compounds were obtained from previous research (already published, [6]) and used simultaneously to carry out this study. Spectra data as well as melting points are available as supplementary materials in a previous paper [6].

### 4.2. Biological Evaluation

#### 4.2.1. Cell Lines

Human biphenotypic B myelomonocytic leukemia MV4-11, murine normal fibroblast BALB/3T3 cells were obtained from the American Type Culture Collection (USA); and human lung carcinoma A549, human breast cancer MCF-7 and human prostate adenocarcinoma PC-3 were obtained from the European Collection of Authenticated Cell Cultures (UK). All the cell lines were maintained at the Hirszfeld Institute of Immunology and Experimental Therapy, PAS, Wroclaw, Poland.

MV4-11 and PC-3 cells were cultured in RPMI 1640 medium (HIIET PAS, Poland) with 1.0 mM sodium pyruvate (only MV4-11) and 10% fetal bovine serum (FBS) (all from Merck, Darmstadt, Germany). A549 cells were cultured in RPMI 1640 + Opti-MEM (1:1) ((HIIET PAS, Poland and Gibco, UK) supplemented with 5% fetal bovine serum (Merck, Darmstadt, Germany). The MCF-7 cells were cultured in Eagle medium (HIIET PAS, Poland) supplemented with 8 mg/mL of insulin and 1% of MEM NON-Essential amino acid (all Merck, Darmstadt, Germany). Normal fibroblasts BALB/3T3 cells were cultured in DMEM medium (Gibco, UK) supplemented with 10% fetal bovine serum and 1.0 mM sodium pyruvate (all from Merck, Darmstadt, Germany). All culture media were supplemented with 2 mM L-glutamine (Merck, Darmstadt, Germany), 100 units/ml penicillin, (Polfa Tarchomin S.A., Poland) and 100 µg/mL streptomycin (Merck, Darmstadt, Germany). All cell lines were grown at 37 °C in a 5% CO_2_ humidified atmosphere.

#### 4.2.2. Determination of Antiproliferative Activity

The solutions of the tested compounds were prepared by dissolving the substances in DMSO to the desired concentration (25 mM). Then, the tested compounds were diluted in a culture medium to reach the final concentrations. Before addition of the tested compounds (24 h prior), the cells were plated in 96-well plates (Sarstedt, Germany) at a density of 1 × 10^4^, 0.75 × 10^4^ (MCF-7) or 0.5 × 10^4^ (A549) cells per well. The assay was performed after 72 h of exposure to 100, 10, 1 and 0.1 µM concentration of the tested agents. The in vitro cytotoxic effect of all agents was examined using the MTT (MV4-11) or SRB assay, as described previously [36]. Briefly, the MTT method showed the cells viability, and the number of cells that converted the water soluble MTT reagent (3-(4,5-dimethylthiazol-2-yl)-2,5-diphenyltetrazolium bromide) to an insoluble formazan product. The SRB method determined the amount of cellular proteins. The concentration of the colorimetric probe (formazan in MTT or sulforhodamine B in SRB method) was determined by an optical density measurement using a standard microplate reader for 96-well plates. After optical density measurement, using the data of the mean absorbances of control cells (AbCC), the absorbances of cells treated by the tested compound (AbTr) (for each concentration separately) and the absorbance of the cultured medium (AbCM) the % of cell proliferation inhibition was calculated according to the following formula:

$$\%\;inh.\;cells\;proliferation=100-\frac{AbTr-AbCM}{AbCC-AbCM}\times 100$$

From the obtained proliferation inhibition values, effect-concentration curves of the compound used were plotted. The results were calculated as an IC_50_ (inhibitory concentration 50%) of the concentration of the tested agent, which is cytotoxic for 50% of the cancer cells. IC_50_ values were calculated for each experiment separately using the Prolab-3 system based on Cheburator 0.4 software [36] and mean values (±SD) are presented. Each compound in each concentration was tested in triplicate in a single experiment, which was repeated 3–5 times.

#### 4.2.3. Cell Cycle Analysis

The MV4-11, A549 and PC-3 cells were seeded on 24-well plates (Sarstedt, Germany) for a final volume of 1.5 mL. The cells were exposed to the tested compounds **2**, **2a**–**d** and **5** at concentrations of about IC_50_ for 24 and 72 h. After incubation, the cells were collected and 1 × 106 of cells were washed twice in cold PBS and fixed for 24 h in 70% ethanol at −20 °C, the cells were washed twice in PBS and incubated with RNAse (8 μg/mL, Fermentas, Germany) at 37 °C for 1 h. The cells were stained for 30 min. with propidium iodide (50 μg/mL, Sigma Aldrich, Germany) at 4 °C and the cellular DNA content was analyzed by flow cytometry using BD LSRFortessa cytometer (BD Bioscience, San Jose, CA, USA). Compounds at each concentration were tested at least three times independently. The obtained results were analyzed using Flowing software 2.5.1 (Cell Imaging Core, Turku Centre for Biotechnology, University of Turku and Åbo Akademi University, Turku, Finland).

#### 4.2.4. Apoptosis Determination by Annexin V Staining

The MV4-11, A549 and PC-3 cells were seeded on 24-well plates (Sarstedt, Germany) for a final volume of 1.5 mL. The cells were exposed to the tested compounds **2**, **2a**–**d** and **5** at concentrations of about 1.5×IC_50_ for 24 and 72 h. After incubation, the cells were collected and 2 × 105 of cells were washed twice with PBS. APC-Annexin V (BD Pharmingen) was dissolved in a concentration of 1 mg/mL in a binding buffer (Hepes buffer: 10 mM HEPES/NaOH, pH 7.4, 150 mM NaCl, 5 mM KCl, 1 mM MgCl_2_, 1.8 mM CaCl_2_, (HIIET, Poland) and the cells were suspended in 200 µL of this 1 mg/mL solution (freshly prepared each time). After 15 min of incubation in the dark at room temperature, the propidium iodide (PI) solution (1 mg/mL) was added prior to the analysis to give a final concentration of 0.1 mg/mL. Data acquisition was performed by flow cytometry using a BD LSRFortessa cytometer. The compounds were tested at least three times independently. Results were analyzed using Flowing software 2.5.1. The data were displayed as a two-color dot plot with an APC-Annexin V vs. PI. Double-negative cells were live cells, PI+/Annexin V+ were late apoptotic, PI-/Annexin V+ were early apoptotic cells and PI+/Annexin V- were necrotic cells.

#### 4.2.5. Caspase-3/7 Activity Determination

The MV4-11, A549 and PC-3 cells were seeded on 48-well plates for a final volume of 1 mL. The cells were exposed to the tested compounds at concentrations of about 1.5 × IC_50_ or campthothecin (0.05–0.1 µg/mL) as a positive control, for 24 and 72 h. After incubation, the cells were collected and centrifuged (5 min., 4 °C, 250× *g*). Cells were suspended in 50 µL of ice-cold lysis buffer (50 mM HEPES, 10% (w/v) sucrose, 150 mM NaCl, 2 mM EDTA and 1% (v/v) Triton X-100, pH 7.3, HIIET, Poland) and incubated 30 min. at 40 °C. After the incubation, 40 µL of each sample was transferred to a white, 96-well plate (Corning, CA, USA) containing 160 µL of the reaction buffer (20 mM HEPES, 10 % sucrose, 100 mM NaCl, 1 mM EDTA, 10 mM DTT, 0.02 % Trition X-100, pH 7.3) (HIIET, Wroclaw, Poland) with 9 µM AC-DEVD-AMC (Cayman Chemical) fluorogenic substrate (λ_ex_ = 360 nm, λ_em_ = 460 nm). The fluorescence increase correlated with the caspase-3/7 level was continuously recorded at 37 °C for 120 min using a Biotek Synergy H4 (Biokom, Warsaw, Poland). Compounds were tested in duplicates in a single experiment and each experiment was repeated at least three times independently. Results were normalized to the number of cells in each well and are reported as mean relative caspase-3/7 activity compared to the untreated control sample ± SD.

### 4.3. Lipophilicity

The RP-TLC chromatographic procedure used to determine the experimental logP value is included in the Supplementary Materials. To calculate the theoretical values of logP partition coefficients, the Chem Draw software (Perkin Elmer Informatics) was used to determine the Clog P values as well as transform the structures of the studied molecules into a simplified molecular-input line-entry system (SMILES). Based on SMILES codes, and using freely available web tools, such as pkCSM (Biosig Lab—University of Melbourne) and ALOGPS 2.1 (Virtual Computational Chemistry Laboratory), further theoretical logP values were calculated [28].

### 4.4. In Silico Analysis

Prediction of the pharmacokinetic profile of the tested compounds, including the calculation of molecular and physicochemical descriptors, as well as the assessment of similarity to drugs, was performed using the pkCSM-Biosig Lab—University of Melbourne (http://biosig.unimelb.edu.au) server.

### 4.5. Statistical Analysis

All datasets were analyzed using t-test. P-values lower than 0.05 were considered as statistically significant. Statistical analysis was performed using Statsoft Statistica 10 software.

## Figures and Tables

**Table 1:** Growth inhibition (IC_50_, µM) efficiency of reference compounds (betulin **1**, cisplatin and doxorubicin) and betulin derivatives on leukemia cell line; n/s-not studied due to insolubility in DMSO.

	**R_2_**	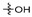			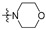		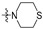		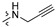			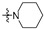			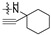	
R_1_																
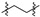		**2** (23.18)			**2b** (3.94)		**2d** (3.16)		**2a** (2.03)			**2c** (5.78)			**2e** (52.54)	
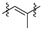		**3** (19.95)			**3b** (8.90)		**3d** (4.23)		**3a** (12.52)			3c (n/s)			3e (22.18)	
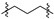		**4** (24.01)			**4b** (6.27)		**4d** (7.04)		**4a** (4.08)			4c (n/s)			4e (22.04)	
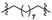		**5** (5.01)			**5b** (6.97)		5d (43.56)		5a (21.03)			5c (n/s)			5e (100.00)	
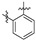		**6** (34.38)			**6b** (15.03)		6d (14.31)		6a (24.04)			6c (100.00)			6e (54.61)	
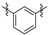		**7** (23.54)			**7b** (47.58)		7d (72.24)		7a (79.68)			7c (n/s)			7e (100.00)	
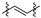		**8** (15.25)														
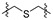		**9** (26.40)														
		Betulin **1** (18.16)														
		Cisplatin (1.83)														
		Doxorubicin (0.01)														
	
Scale:		1	2	5		10	15	18.16		25	30		40	45		50

The color scheme visualizes active compounds in green and inactive ones in red. Color saturation corresponds to their degree of activity as indicated on the scale.

**Table 2:** Comparison of cytotoxicity (IC_50_) of the tested compounds against BALB/3T3 cell line depending on the dicarboxylic linker and amine present in their structure.

	**R_2_**	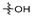			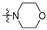			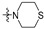			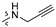			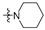	
R_1_															
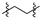		**2** (53.37)			**2b** (25.04)			**2d** (50.00)			**2a** (3.10)			**2c** (32.71)	
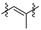		**3** (37.17)			**3b** (24.69)			**3d** (50.00)							
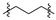		**4** (37.90)			**4b** (36.76)			**4d** (50.00)			**4a** (5.02)				
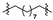		**5** (23.44)			**5b** (20.36)										
		Betulin **1** (31.79)													
		Doxorubicin (0.18)													

Scale:		1	2	5		10	15		25	31.79		40	45		50

The color scheme visualizes active compounds in green and inactive ones in red. Color saturation corresponds to their degree of activity as indicated on the scale.

**Table 3:** Comparison of cytotoxicity (IC_50_, µM) of the tested compounds against A549cell line depending on the dicarboxylic linker and amine present in their structure.

	R_2_	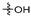			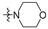			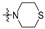			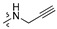			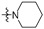	
R_1_															
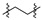		**2** (35.30)			**2b** (8.03)			**2d** (32.01)			**2a** (19.15)			**2c** (6.98)	
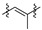		**3** (28.70)			**3b** (7.42)			**3d** (6.78)							
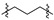		**4** (32.48)			**4b** (8.47)			**4d** (40.20)			**4a** (29.10)				
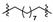		**5** (4.91)			**5b** (12.58)										
		Betulin **1** (15.51)													
		Doxorubicin (0.04)												**A549**	

Scale:		1	2	5		10	15.51		25	35		40	45		50

The color scheme visualizes active compounds in green and inactive ones in red. Color saturation corresponds to their degree of activity as indicated on the scale.

**Table 4:** Comparison of cytotoxicity (IC_50_, µM) of the tested compounds against MCF-7 cell line depending on the dicarboxylic linker and amine present in their structure.

	**R_2_**	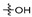			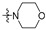		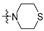			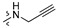			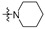		
R_1_															
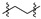		**2** (53.64)			**2b** (19.27)		**2d** (50.00)			**2a** (29.79)			**2c** (22.18)		
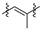		**3** (38.22)			**3b** (12.27)		**3d** (34.05)								
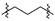		**4** (43.93)			**4b** (21.61)		**4d** (50.00)			**4a** (50.00)					
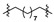		**5** (12.27)			**5b** (19.54)										
		Betulin **1** (38.82)													
		Doxorubicin (0.08)											**MCF-7**		

Scale:		1	2	5		10		15	25		35	38.82		45	50

The color scheme visualizes active compounds in green and inactive ones in red. Color saturation corresponds to their degree of activity as indicated on the scale.

**Table 5:** Comparison of cytotoxicity (IC_50_, µM) of the tested compounds against PC-3 cell line depending on the dicarboxylic linker and amine present in their structure.

	R_2_	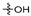			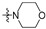			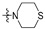			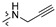			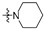	
R_1_															
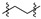		**2** (68.55)			**2b** (17.86)			**2d** (50.00)			**2a** (4.47)			**2c** (30.88)	
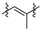		**3** (34.42)			**3b** (24.84)			**3d** (46.11)							
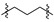		**4** (36.19)			**4b** (22.69)			**4d** (50.00)			**3a** (15.29)				
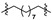		**5** (19.77)			**5b** (22.46)										
		Betulin **1** (32.46)													
		Doxorubicin (0.43)												**PC-3**	

Scale:		1	2	5		10	15		25	32.46		40	45		50

The color scheme visualizes active compounds in green and inactive ones in red. Color saturation corresponds to their degree of activity as indicated on the scale.

**Table 6:** The SI index = IC_50_ for normal cell line (BALB/3T3)/IC_50_ for respective cancerous cell line. A beneficial SI > 1.0 indicates a drug with efficacy against tumor cells greater than toxicity against normal cells.

Compound	Cell Lines/Calculated Selectivity Index SI			
	MV4-11	A549	MCF-7	PC-3
**2**	2.3	1.5	1.0	0.8
**2a**	1.5	0.2	0.1	0.7
**2b**	6.4	3.1	1.3	1.4
**2c**	5.7	4.7	1.5	1.1
**2d**	15.8	1.6	1.0	1.0
**3**	1.9	1.3	1.0	1.1
**3b**	2.8	3.3	2.0	1.0
**3d**	11.8	7.4	1.5	1.1
**4**	1.6	1.2	0.9	1.0
**4a**	1.2	0.2	0.1	0.3
**4b**	5.9	4.3	1.7	1.6
**4d**	7.1	1.2	1.0	1.0
**5**	4.7	4.8	1.9	1.2
**5b**	2.9	1.6	1.0	0.9
**Betulin 1**	1.8	2.0	0.8	1.0
**Doxorubicin**	18.0	4.5	2.3	0.4

**Table 7:** Comparison of antiproliferative and caspase-3/7 activities of tested compounds on A549 and PC-3 cell lines.

	A549			PC-3		
	Induction of Apoptosis	Caspase 3/7 Activity *	Antiproliferative Activity **	Induction of Apoptosis	Caspase 3/7 Activity *	Antiproliferative Activity **
**2**	+	+	−	+	+	−
**2a**	−	−	−	+	−	+
**2b**	−	+	+	+	+	+
**2c**	−	+	+	−	−	−
**2d**	−	−	−	−	−	−
**5**	+	+	+	+	+	+

* plus mark represents statistically important elevated activity of caspase 3/7 compared to control; ** plus mark represents antiproliferative IC_50_ lower than reference compound used (betulin **1**).

**Table 8:** The experimental values of lipophilicity for tested compounds.

Compound	R_M0_	−b	*r*	log P_TLC_
**2**	5.910	0.071	0.982	8.191
**2a**	4.863	0.059	0.997	6.796
**2b**	5.024	0.059	0.990	7.011
**2c**	5.728	0.065	0.994	7.949
**2d**	5.898	0.068	0.987	8.176
**2e**	6.027	0.070	0.993	8.347
**3**	5.410	0.065	0.995	7.525
**3a**	5.326	0.063	0.996	7.413
**3b**	5.086	0.060	0.991	7.093
**3c**	5.650	0.064	0.993	7.845
**3d**	5.711	0.066	0.993	7.926
**3e**	6.098	0.070	0.997	8.441
**4**	5.250	0.062	0.996	7.312
**4a**	5.158	0.061	0.993	7.189
**4b**	4.961	0.057	0.995	6.926
**4c**	5.012	0.055	0.989	6.994
**4d**	5.008	0.057	0.998	6.989
**4e**	5.920	0.068	0.995	8.205
**5**	5.949	0.068	0.998	8.247
**5a**	5.998	0.069	0.995	8.308
**5b**	5.572	0.062	0.995	7.740
**5c**	5.768	0.062	0.988	8.002
**5d**	5.044	0.055	0.983	7.037
**5e**	6.093	0.068	0.991	8.435
**6**	5.979	0.071	0.988	8.283
**6a**	6.016	0.072	0.986	8.332
**6b**	5.979	0.070	0.983	8.284
**6c**	5.875	0.063	0.986	8.145
**6d**	6.255	0.072	0.981	8.652
**6e**	6.412	0.074	0.985	8.861
**7**	5.817	0.066	0.996	8.067
**7a**	5.839	0.067	0.990	8.096
**7b**	5.153	0.058	0.959	7.182
**7c**	5.773	0.063	0.985	8.008
**7d**	5.980	0.066	0.986	8.284
**7e**	5.746	0.061	0.987	7.972
**8**	5.129	0.063	0.995	7.150
**9**	6.087	0.073	0.988	8.427

**Table 9:** The pharmacokinetic parameters of tested compounds (pkCSM-Biosig Lab—University of Melbourne http://biosig.unimelb.edu.au; accessed on 15 September 2022).

Compound	MW	nHBA	nHBD	nROT	TPSA (Å^2^)	Caco-2 Perm. (log Papp, cm/s)
**2**	542.80	4	2	6	83.83	0.633
**2a**	579.87	4	2	7	75.63	0.703
**2b**	611.91	5	1	6	76.07	0.713
**2c**	609.94	4	1	6	66.84	0.611
**2d**	627.97	5	1	6	66.84	0.627
**2e**	647.99	4	2	7	75.63	0.654
**3**	554.81	4	2	5	83.83	0.653
**3a**	591.88	4	2	6	75.63	0.723
**3b**	623.92	5	1	5	76.07	0.733
**3c**	621.95	4	1	5	66.84	0.631
**3d**	639.98	5	1	5	66.84	0.647
**3e**	660.00	4	2	6	75.63	0.674
**4**	556.83	4	2	7	83.83	0.609
**4a**	593.89	4	2	8	75.63	0.708
**4b**	625.94	5	1	7	76.07	0.719
**4c**	623.96	4	1	7	66.84	0.617
**4d**	642.00	5	1	7	66.84	0.633
**4e**	662.01	4	2	8	75.63	0.659
**5**	612.94	4	2	11	83.83	0.461
**5a**	650.00	4	2	12	75.63	0.569
**5b**	682.04	5	1	11	76.07	0.580
**5c**	680.07	4	1	11	66.84	0.478
**5d**	698.11	5	1	11	66.84	0.494
**5e**	718.12	4	2	12	75.63	0.521
**6**	590.85	4	2	5	83.83	0.683
**6a**	627.91	4	2	6	75.63	0.668
**6b**	659.95	5	1	5	76.07	0.699
**6c**	657.98	4	1	5	66.84	0.614
**6d**	676.02	5	1	5	66.84	0.630
**6e**	696.03	4	2	6	75.63	0.620
**7**	590.85	4	2	5	83.83	0.714
**7a**	627.91	4	2	6	75.63	0.724
**7b**	659.95	5	1	5	76.07	0.736
**7c**	657.98	4	1	5	66.84	0.652
**7d**	676.02	5	1	5	66.84	0.667
**7e**	696.03	4	2	6	75.63	0.675
**8**	540.79	4	2	5	83.83	0.661
**9**	574.87	5	2	7	83.83	0.684

## Data Availability

Not applicable.

## Supplementary Materials

The following supporting information can be downloaded at: https://www.mdpi.com/article/10.3390/ijms24010196/s1. Table S1. The literature lipophilicity (logP_lit_) and experimental (R_M0_) values of reference compounds; Table S2. The theoretical values of lipophilicity for tested compounds; Table S3. The correlation matrix for theoretically obtained lipophilicity parameters of tested compounds; Table S4. The correlation between the molecular descriptors and the R_M0_ values for betulin derivatives; Table S5. The correlation between the anticancer activity (IC_50_) and the R_M0_ values for betulin derivatives. Reference [37] is cited in the supplementary materials.
