# Supplementary material for: Betulin Acid Ester Derivatives Inhibit Cancer Cell Growth by Inducing Apoptosis through Caspase Cascade Activation: A Comprehensive In Vitro and In Silico Study
Source: Int J Mol Sci. 2022 Dec 22;24(1):196. doi: 10.3390/ijms24010196 (PMC9820118; doi:10.3390/ijms24010196)
Supplement: Supplementary file 1 [file ijms-24-00196-s001.zip › ijms-2083203-supplementary.pdf]

Supplementary Materials

Novel betulin acid ester derivatives inhibit cancer cell growth by inducing apoptosis through caspase cascade activation: a comprehensive *in vitro* and *in silico* study

LIST OF CONTENTS

Chromatographic analysis by RP-TLC

Table S1. The literature lipophilicity ($logP_{lit}$) and experimental (R_{M0}) values of reference compounds

Table S2. The theoretical values of lipophilicity for tested compounds

Table S3. The correlation matrix for theoretically obtained lipophilicity parameters of tested compounds

Table S4. The correlation between the molecular descriptors and the R_{M0} values for betulin derivatives

Table S5. The correlation between the anticancer activity (IC_{50}) and the R_{M0} values for betulin derivatives

Chromatographic analysis by RP-TLC

Chromatographic analysis by RP-TLC was performed an aluminum backed silica gel RP-18 $F_{254}S$ plates (Merck, Darmstadt, Germany). Reference compounds [acetanilide, prednisone, 4-bromoacetophenone, benzophenone, anthracene, dibenzyl, dichlorodiphenyltrichloroethane (DDT), 9-phenylanthracene] and tested betulin derivatives were dissolved in chloroform to obtain a concentration of 2 mg/mL. The prepared solutions were spotted on the plates in the amount of 2 μL. In the next step, the chromatographic chambers were saturated 30 min of the appropriate mobile phases consisting of a mixture of acetone (Merck, Darmstadt, Germany) and aqueous solution of buffer Tris [(tris-hydroxymethyl)aminomethane] (Fluka, Loughborough, 0.2 M, pH 7.4). The amount of acetone in the mobile phase were in the range 60-90% in 5% increments. The chromatograms were developed for each compound in triplicate at room temperature. The spots of betulin derivatives were visualized by spraying the plates with the mixture of ethanol and concentrated sulfuric acid (10:1, v/v) and heating at 110°C for 3 min.

The retardation factor (R_f) were calculated according to the equation below:

$$R_f = \frac{a}{b}$$

(a-the distance from the start line to the spot center, b-the distance from the start line to the mobile phase front)

The R_M values were calculated by the Bate-Smith and Westall equation:

$$R_M = \log\left(\frac{1}{R_f} - 1\right)$$

The R_{M0} parameters were obtained by extrapolating on the acetone content to zero, according to the Soczewiński-Wachtmeister equation:

$$R_M = R_{M0} + bC$$

(*C*-the percentage concentration of acetone in the mobile phase, b-the slope of the regression plot).

The calibration curve was determined using eight reference compounds. $LogP_{lit}$ values (from literature) of these compounds are in the range 1.21–6.38 [37]. The R_{M0} values for the reference compounds were determined under the same conditions as for betulin derivatives (Table S1).

Table S1. The literature lipophilicity ($logP_{lit}$) and experimental (R_{M0}) values of reference compounds

**Compound**	**$LogP_{lit}$**	**R_{M0}**	***b***	***r***
acetanilide	1.21	0.56	-0.01	0.958
prednisone	1.62	0.80	-0.02	0.939
4-bromoacetophenone	2.43	1.88	-0.02	0.995
benzophenone	3.18	2.32	-0.03	0.998
anthracene	4.45	3.10	-0.0344	0.980
dibenzyl	4.49	3.54	-0.04	0.996
9-phenyloanthracene	6.01	3.92	-0.04	0.996
DDT	6.38	4.55	-0.05	0.998

The experimental values of lipophilicity (log P_{TLC}) for compounds **2**, **2a-e**, **3**, **3a-e**, **4**, **4a-e**, **5**, **5a-e**, **6**, **6a-e**, **7**, **7a-e**, **8** and **9** were calculated using the calibration curve (the correlation between the log P_{lit} and R_{M0} for reference compounds).

$$\log P_{TLC} = 1{,}3331 R_{M0} + 0{,}3127 \; (r = 0.9894, SD = 0.282)$$

Table S2. The theoretical values of lipophilicity for tested compounds

Compd	C log P[a]	Log P[b]	ALOGPs	AC log P	ALOGP	MLOGP	XLOGP2	XLOGP3
**2**	9.24	7.41	5.39	5.90	6.51	5.83	7.92	8.29
**2a**	9.20	7.08	5.77	5.78	7.31	5.87	7.98	8.15
**2b**	9.28	7.19	5.28	6.02	6.01	5.37	7.46	7.85
**2c**	10.07	8.34	6.52	7.23	7.24	6.31	8.72	9.07
**2d**	10.01	7.90	5.68	6.52	6.75	6.14	8.62	8.65
**2e**	11.22	8.78	6.40	7.27	9.02	6.73	9.35	9.75
**3**	9.88	7.58	5.76	6.02	7.36	5.92	8.36	9.00
**3a**	9.78	7.24	5.95	5.90	8.15	5.96	8.42	8.86
**3b**	9.96	7.35	5.54	6.14	6.85	5.45	7.89	8.56
**3c**	11.06	8.51	6.74	7.35	8.08	6.39	9.16	9.78
**3d**	10.70	8.07	5.92	6.64	7.60	6.22	9.06	9.35
**3e**	11.80	8.95	6.62	7.39	9.87	6.81	9.79	10.46
**4**	9.62	7.80	5.60	6.36	6.97	6.01	8.28	8.65
**4a**	9.48	7.47	5.92	6.24	7.76	6.05	8.34	8.51
**4b**	9.50	7.58	5.56	6.49	6.47	5.54	7.81	8.21
**4c**	10.30	8.73	6.80	7.70	7.69	6.48	9.08	9.42
**4d**	10.24	8.29	5.96	6.98	7.21	6.31	8.98	9.00
**4e**	11.50	9.17	6.60	7.73	9.48	6.90	9.71	10.11
**5**	11.23	9.36	6.83	8.22	8.79	6.72	9.93	10.63
**5a**	10.76	9.03	6.93	8.10	9.59	6.73	9.98	10.49
**5b**	10.61	9.14	6.95	8.34	8.29	6.22	9.46	10.19
**5c**	11.40	10.29	7.87	9.55	9.52	7.15	10.72	11.41
**5d**	11.34	9.85	7.22	8.84	9.03	6.99	10.62	10.98
**5e**	12.79	10.73	7.46	9.59	11.30	7.55	11.56	12.09
**6**	10.44	8.56	6.23	7.32	7.96	6.82	9.89	10.04
**6a**	10.61	8.22	6.35	7.20	8.76	6.85	9.94	9.91
**6b**	10.17	8.33	5.94	7.44	7.46	6.33	9.42	9.60

**6c**	11.20	9.49	7.02	8.65	8.69	7.27	10.68	10.82
**6d**	10.90	9.05	6.42	7.94	8.20	7.10	10.58	10.40
**6e**	12.63	9.93	7.01	8.69	10.47	7.68	11.31	11.51
**7**	11.32	8.56	6.21	7.32	7.96	6.82	9.89	10.04
**7a**	10.91	8.22	6.32	7.20	8.76	6.85	9.94	9.91
**7b**	10.17	8.33	5.94	7.44	7.46	6.33	9.42	9.60
**7c**	11.20	9.49	6.96	8.65	8.69	7.27	10.68	10.82
**7d**	10.90	9.05	6.43	7.94	8.20	7.10	10.58	10.40
**7e**	12.93	9.93	7.03	8.69	10.47	7.68	11.31	11.51
**8**	9.57	7.19	5.54	5.54	6.91	5.74	8.22	8.62
**9**	9.17	7.36	5.55	5.66	7.06	5.83	8.18	9.13

[a] Chem Draw

[b] pkCSM

Table S3. The correlation matrix for theoretically obtained lipophilicity parameters of tested compounds

	**C log P**[a]	**Log P**[b]	**ALOGPs**	**AC log P**	**ALOGP**	**MLOGP**	**XLOGP2**	**XLOGP3**
**C log P**[a]	1.0000	0.897	0.813	0.839	0.910	0.901	0.902	0.918
**Log P**[b]		1.0000	0.931	0.981	0.846	0.902	0.929	0.956
**ALOGPs**			1.0000	0.937	0.842	0.833	0.853	0.903
**AC log P**				1.0000	0.791	0.869	0.913	0.928
**ALOGP**					1.0000	0.869	0.863	0.903
**MLOGP**						1.0000	0.977	0.935
**XLOGP2**							1.0000	0.971
**XLOGP3**								1.0000

Table S4. The correlation between the molecular descriptors and the R_{M0} values for betulin derivatives

Compound	Molecular descriptors	Equation	*r*
**2, 2a-e**	*MW*	R_{M0} = 0.0003 MW^2-0.3541 *MW*+109.42	0.774
	HBA	*HBA* = -0.0893 R_{M0}^2+0.6821 R_{M0}+3.3	0.516
	HBD	*HBD* = 4.4729 R_{M0}^2-48.529 R_{M0}+132.09	0.807
	nROT	*nROT* = 4.4863 R_{M0}^2-48.942 R_{M0}+138.81	0.880
	TPSA	*TPSA* = 27.066 R_{M0}^2-295.36 R_{M0}+873.86	0.426
	Caco-2 perm.	*Caco-2 perm* = 0.1483 R_{M0}^2-1.682 R_{M0}+5.3951	0.919
**3, 3a-e**	*MW*	R_{M0} = 0.0002 MW^2-0.1973 *MW*+63.379	0.833
	HBA	*HBA* = 0,8774 R_{M0}^2-10.299 R_{M0}+34.375	0.383
	HBD	*HBD* = 0.5856 R_{M0}^2-6.2428 R_{M0}+18.05	0.237
	nROT	*nROT* = 1.5987 R_{M0}^2-17.363 R_{M0}+52.288	0.516
	TPSA	*TPSA* = 17.104 R_{M0}^2-197.36 R_{M0}+640.85	0.450
	Caco-2 perm.	*Caco-2 perm* = 0.2264 R_{M0}^2-2.605 R_{M0}+8.1373	0.872
**4, 4a-e**	*MW*	R_{M0} = 0.0002 MW^2-0.275 *MW*+87.53	0.849
	HBA	*HBA* = 3.6342 R_{M0}^2-40.383 R_{M0}+115.71	0.755
	HBD	*HBD* = -4.6519 $R_{M0}{}^2$+51.808 R_{M0}-141.67	0.964
	nROT	*nROT* = -0.8386 R_{M0}^2+10.14 R_{M0}-22.654	0.702
	TPSA	*TPSA* = -48.933 R_{M0}^2+539.91 R_{M0}+1405.5	0.690
	Caco-2 perm.	*Caco-2 perm* = 0.1538 R_{M0}^2-1.6878 R_{M0}+5.2598	0.273
**5, 5a-e**	*MW*	R_{M0} = 0.0001 MW^2-0.1417 *MW*+53.719	0.446
	HBA	*HBA* = -0.694 R_{M0}^2+6.5685 R_{M0}-10.421	0.874
	HBD	*HBD* = 2.3844 R_{M0}^2-25.407 R_{M0}+68.484	0.929
	nROT	*nROT* = 2.4518 R_{M0}^2-26.44 R_{M0}+82.017	0.825
	TPSA	*TPSA* = -1.2005 R_{M0}^2+23.362 R_{M0}+20.229	0.600
	Caco-2 perm.	*Caco-2 perm* = -0.0802 R_{M0}^2+0.9021 R_{M0}-2.0073	0.205
**6, 6a-e**	*MW*	R_{M0} = 0.0001 MW^2-0.1255 *MW*+45.065	0.889
	HBA	*HBA* = -10.306 R_{M0}^2+127.11 R_{M0}-387.17	0.550
	HBD	*HBD* = -0.1016 R_{M0}^2+1.9653 R_{M0} -6.6933	0.266
	nROT	*nROT* = 2.3255 R_{M0}^2-27.38 R_{M0}+85.754	0.501
	TPSA	*TPSA* = -43.991 R_{M0}^2+538.57 R_{M0}+1572.7	0.202
	Caco-2 perm.	*Caco-2 perm* = -0.7613 R_{M0}^2+9.2923 R_{M0}-27.675	0.717
**7, 7a-e**	*MW*	R_{M0} = 7E-05 MW^2-0.085 *MW*+33.17	0.299

	HBA	*HBA* = 7.842 R_{M0}^2+87.402 R_{M0}+247.15	0.973
	HBD	*HBD* = -5.1737 R_{M0}^2+57.778 R_{M0}+159.36	0.633
	nROT	*nROT* = -3.9003 R_{M0}^2+43.466 R_{M0}-115.41	0.483
	TPSA	*TPSA* = -37.763 R_{M0}^2+412.03 R_{M0}+1044.6	0.416
	Caco-2 perm.	*Caco-2 perm* = 0.0609 R_{M0}^2-0.74 R_{M0}+2.9319	0.574

Table S5. The correlation between the anticancer activity (IC_{50}) and the R_{M0} values for betulin derivatives

Compound	Cell line	Equation	*r*
**2, 2a-e**	MV4-11	IC_{50} = 5.9737 R_{M0}^2-37.473 R_{M0}+55.657	0.913
**2, 2a-e**	A549	IC_{50} = 134.01 R_{M0}^2-1432.5 R_{M0}+3818.7	0.9750
**2, 2a-d**	MCF-7	IC_{50} = 142.35 R_{M0}^2-1515.4 R_{M0}+4035.9	0.979
**2, 2a-d**	PC-3	IC_{50} = 66.594 R_{M0}^2-673.01 R_{M0}+1708.9	0.929
